# Online-Ressourcen zu Sprunggelenkdistorsionen: Eine deutschsprachige Webanalyse

**DOI:** 10.1007/s00113-024-01526-x

**Published:** 2025-01-16

**Authors:** Sophie Maria Tengler, Mark Lenz, Gunther O. Hofmann, Marianne Rosenthal, Klaus E. Roth, Lena Mohr, Hazibullah Waizy, Kajetan Klos

**Affiliations:** 1 Klinik für Unfall‑, Hand und Wiederherstellungschirurgie, Universitätsklinikum Jena, Friedrich-Schiller-Universität Jena, Am Klinikum 1, 07747 Jena, Deutschland; 2 Friedrich-Schiller-Universität Jena, 07743 Jena, Deutschland; 3 Meliva Gelenkzentrum Rhein-Main, 65239 Hochheim, Deutschland; 4 Universitätsmedizin Mainz, 55131 Mainz, Deutschland; 5 Orthoprofis, 30159 Hannover, Deutschland

**Keywords:** Patientenratgeber, Digitale Gesundheit, Internetseiten, Qualität, Lesbarkeit, Ankle sprain, Web analysis, Websites, Quality, Readability

## Abstract

**Hintergrund:**

Mit dem Internet als Hauptinformationsquelle für Gesundheitsinhalte ist die Qualität von Webseiten mit medizinischen Informationen von hoher Bedeutung.

**Ziel der Arbeit:**

Diese Studie untersucht 250 Webseiten über die akute Sprunggelenkdistorsion (ASD) als eine der häufigsten Erkrankungen des Bewegungssystems auf ihre Qualität und Lesbarkeit. Anhand der Ergebnisse wurde ein eigener Ratgeber erstellt.

**Methode:**

Mithilfe eines 25-Item-Scores sowie des EQIP36-Scores für medizinische Informationsmaterialien wurde die Qualität der Webseiten eingeschätzt. Das Leseniveau konnte anhand des Flesch-Kincaid-Index und der errechneten Lesbarkeit ermittelt werden. Die besten 3 Internetseiten wurden in einer Benutzerumfrage evaluiert.

**Ergebnisse:**

Von 250 erfassten Webseiten konnten 77 in die Studie eingeschlossen werden. Die Qualität dieser variiert erheblich; keine erfüllte alle Anforderungen. Quellen des Gesundheitssystems zeigten eine höhere Qualität, während kommerziell betriebene Seiten eine unterdurchschnittliche inhaltliche Vollständigkeit aufwiesen. Lediglich 14 % der Webseiten erreichten das empfohlene Leseniveau. Es wurde ein signifikanter Zusammenhang zwischen inhaltlicher Vollständigkeit und geringer Lesbarkeit festgestellt. Die Benutzerumfrage zeigt eine durchwachsene Zufriedenheit. Teilnehmer mit medizinischem Vorwissen waren kritischer als Laien.

**Diskussion:**

Online-Ressourcen zur ASD sind suboptimal und weisen erhebliche Unterschiede auf. Es wurden Mängel in Bezug auf Inhalt, Lesbarkeit und Struktur identifiziert, die die effektive Nutzung durch Patienten einschränken. Herausgeber von Gesundheitsinformationen sollten verstärkt daran arbeiten, die Verständlichkeit und Qualität der bereitgestellten Informationen zu erhöhen.

**Graphic abstract:**

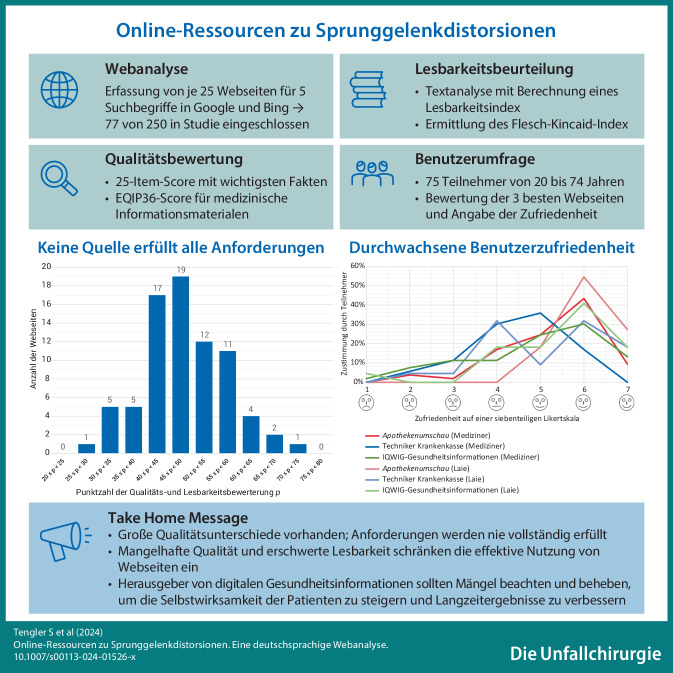

**Zusatzmaterial online:**

Die Online-Version dieses Beitrags (10.1007/s00113-024-01526-x) enthält zusätzliche Tabellen und den Fragebogen der Benutzerumfrage.

## Abstract (en)

**Background:**

With the Internet as the main source of information for health content, the quality of websites with medical information is of high importance.

**Objective:**

This study analysed 250 websites on acute ankle sprain (ASD), one of the most common musculoskeletal disorders, for their quality and readability. Based on the results, a guide for patients was created.

**Method:**

The quality of the websites was assessed using a 25-item content score and the EQIP36 score for medical information material. The reading level could be determined using the Flesch-Kincaid index and the calculated readability. The best three websites were evaluated in a user survey.

**Results:**

Of the 250 websites recorded 77 were included in the study. The quality of these varied significantly, with none achieving the maximum score. Sources in the healthcare system showed higher quality, while commercially influenced sites were below average in terms of completeness of content. Only 14% of the websites reached the recommended reading level. A significant negative correlation was found between completeness of content and readability. The user survey showed a mixed level of satisfaction and participants with previous medical knowledge were more critical than laypersons.

**Conclusion:**

Online resources about ASD are suboptimal and differ considerably. Deficiencies in content, readability and structure were identified, which limit the effective use by patients. Health information publishers should work harder to improve the comprehensibility and quality of the information provided.

Als eine der häufigsten Erkrankungen des Bewegungssystems verursachen akute Sprunggelenkdistorsionen (ASD) weitreichende Folgen für Betroffene und das Gesundheitssystem. Gut informierte Patienten können eine bessere Gesundheitsversorgung und frühzeitige Vorbeugung von Komplikationen erreichen. Mit dem Internet als Hauptinformationsquelle für Gesundheitsinformationen tragen Webseiten mit medizinischen Inhalten essenziell zur Patientenedukation bei. In dieser Arbeit wurden Online-Ressourcen zur ASD erfasst, hinsichtlich ihrer Qualität und Lesbarkeit bewertet und ein eigener Ratgeber [31] erstellt.

## Einleitung

Die ASD ist unter der körperlich aktiven Bevölkerung mit bis zu 40 % die häufigste muskuloskeletale Verletzung [14, 17]. Verstauchungen machen 75 % aller Sprunggelenkverletzungen aus, wobei 85 % davon durch ein Inversionstrauma verursacht sind [16]. Einmal aufgetreten, verdoppelt sich die Wahrscheinlichkeit eines erneuten Traumas [15, 23]. Hohe Behandlungskosten und langfristige Folgen, die sich aus der ersten Verstauchung ergeben, führen zu einer Belastung des Gesundheitssystems [5, 13]. Wird die Verletzung nicht angemessen behandelt, kann dies in bis zu 40 % der Fälle zu einer chronischen Instabilität des oberen Sprunggelenks führen. Dies schränkt Betroffene im Alltag erheblich ein und führt zu einer Reduktion sportlicher Aktivitäten [4, 16, 17]. Eine gute Patientenedukation ist essenziell, um irreversible Schäden zu vermeiden. Nur etwa die Hälfte der Patienten nimmt nach einer akuten Außenbandverletzung ärztliche Hilfe in Anspruch [24]. Mehr als 60 % aller Erwachsenen suchen digital nach gesundheitsbezogenen Auskünften [18, 21]. Mit einem Altersgipfel von ASD bei 15 bis 19 Jahren ist anzunehmen, dass hier der Gebrauch von Online-Ressourcen noch höher geschätzt werden muss [3, 24]. Das Identifizieren qualitativ hochwertiger und seriöser Webseiten ist für Betroffene schwierig und das Angebot sehr vielseitig. Die konsumierten Quellen beeinflussen das weitere Handeln des Patienten und können das Arzt-Patient-Verhältnis sowie die Compliance wesentlich durch hervorgerufene Erwartungen prägen [9]. Ziele dieser Arbeit waren es, die vorhandenen Informationsmaterialien zur ASD auf ihre Qualität und Lesbarkeit zu untersuchen (Stand: Juni 2023) sowie mithilfe einer Benutzerumfrage die effektive Nutzbarkeit durch Patienten einzuschätzen.

## Methode

### Erfassung der Internetseiten.

Knapp 95 % aller Webkonsumenten benutzen die Suchmaschinen Google und Bing, wobei 88 % davon im Mai 2023 Google ausmachten. Mit einem Marktanteil von 96 % steht Google bei der Websuche über mobile Endgeräte an erster Stelle [20]. Zur Festlegung der Suchbegriffe wurde das Programm Google Ads genutzt. Auf Grundlage der Daten vergangener Jahre wurde berechnet, wie viele Klicks und Impressionen pro Suchbegriffkombination durchschnittlich in einem Monat zu erwarten sind. Die gängigsten Suchbegriffe wurden anschließend auf Google Trends im Fünf- sowie im Einjahresverlauf gegeneinander verglichen. Nach Eingabe dieser in einen verlaufsfreien Browser wurden unter Ausschluss aller Werbeanzeigen die je ersten 25 Internetseiten heruntergeladen. Nachdem Duplikate und nichtfunktionierende Links beseitigt wurden, kamen weitere Ausschlusskriterien zum Einsatz. So wurden Webseiten mit beschränktem Zugriff, Video- oder nichtthemenbezogenen Inhalten sowie PowerPoint-Präsentationen und wissenschaftliche Paper nicht miteinbezogen.

### 25-Item-Score.

Basierend auf aktuellen Leitlinien im Umgang mit ASD und Außenbandverletzungen wurde eine Liste der wichtigsten 25 Fakten zusammengestellt (Zusatzmaterial online: eTabelle 1), die von 5 unabhängigen Unfallchirurgen überprüft wurde. Jedes der Items kann mit einem oder keinem Punkt benotet werden.

### EQIP36-Score.

Zur Qualitätsbewertung schriftlicher Patienteninformationen hinsichtlich der Kategorien allgemeine Qualität, Inhalt, Transparenz und Struktur wurde die modifizierte Version des Instruments* Ensuring Quality Information for Patients* (EQIP) genutzt [1, 10, 22, 25]. Die Daten mit den Werten „Ja“, „Nein“ und „Nicht anwendbar“ lassen sich in einen Punktwert bis 100 umrechnen [26]: $\text{EQIP-Score}=\frac{\text{"Ja"} \, \cdot \, 100}{36 \, - \, \text{"Nicht anwendbar"}}.$

### Lesbarkeitsanalyse.

Die Lesbarkeit wurde mithilfe des Textanalysetools Wortliga bewertet [32], welches die Verständlichkeit von Texten, basierend auf dem „Hamburger Verständlichkeitskonzept“, untersucht und neben einem eigenen Lesbarkeitsindex weitere Kennwerte wie den Flesch-Kincaid-Index misst [7, 8]. Ein hoher Punktwert entspricht einer besseren Lesbarkeit. Es folgte die Erfassung von Wort- und Satzanzahl, mittlerer Satzlänge, Buchstaben‑, Zeichen- und Absatzanzahl sowie Sprachmelodie und berechneter Lesedauer.

### Quelle und Urheberschaft der Webseiten.

Alle Webseiten wurden in 2 Schritten kategorisiert. Die Klassifizierung bezüglich der Quelle erfasste, ob das Informationsmaterial von einem kommerziellen Anbieter oder aus dem Sektor des Gesundheitswesens kommt oder mit diesem nicht assoziiert ist [26]. Für die Klassifizierung bezüglich des Urhebers wurden folgende Untergruppen festgelegt: (1) akademische Forschung, (2) Berufsverbände und Fachgesellschaften, (3) Krankenhäuser, (4) Magazine und Zeitschriften, (5) Enzyklopädien, (6) Ärzte und Fußgelenkspezialisten, (7) Apotheken, (8) Krankenkassen, (9) Physiotherapien, (10) Anbieter für Medikamente oder Medizinprodukte (wie Bandagen, Orthesen und Taps), (11) Informationsportale und (12) Sportartikelhersteller.

### Untersuchung auf Bias.

Priorisiert im Suchverlauf können Webseiten mit einem kommerziellen Hintergrund und bezahlter Werbung stehen, was das Informationsbild verzerren und die Qualität beeinflussen kann [27]. Wenn Quellen Werbung für gewinnorientierte Produkte oder Dienstleistungen enthielten, wurden sie als kommerziell verzerrt eingestuft. Ebenso wurde das Vorhandensein von Links zu sozialen Medien geprüft.

### Gesamtwertung.

Die erreichten Punktzahlen des 25-Item-Scores, des EQIP-36-Scores sowie der Lesbarkeit gingen zu je einem Drittel in die Gesamtwertung ein.

### Benutzerumfrage.

Um die ermittelten Ergebnisse in der Realität zu prüfen, wurden die 3 am besten bewerteten Webseiten Benutzern zur Bewertung gegeben. Die Teilnehmer wurden gebeten, Fragen hinsichtlich des Inhalts, der Struktur und der Verständlichkeit zu beantworten, wobei deren medizinisches Vorwissen beachtet wurde (Zusatzmaterial online).

## Ergebnisse

**Abb. 1:**
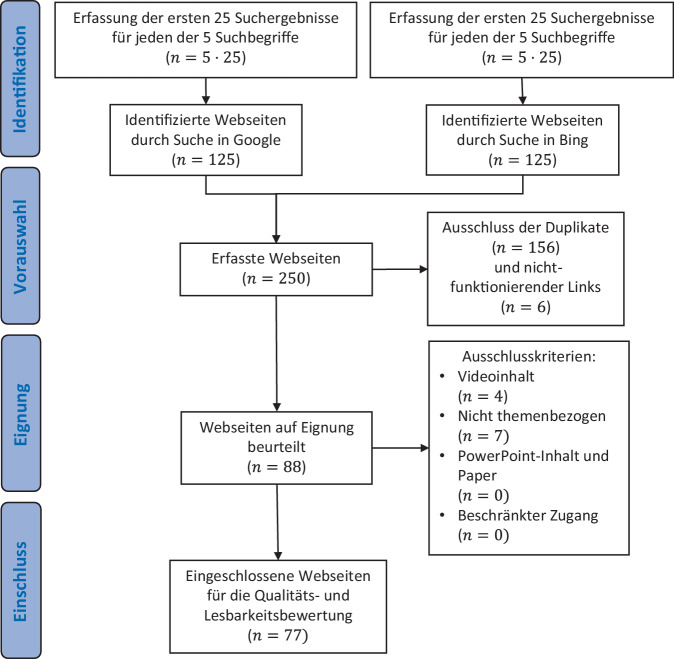
Fließschema der Vorgehensweise zu Erhebung und Selektion der Webseiten, wobei 77 von den 250 erfassten Webseite in die Qualitäts- und Lesbarkeitsbewertung eingeschlossen werden konnten

Eine Prognose für die zu erwartenden Klicks und Impressionen von Suchbegriffen für den Juni 2023 in Deutschland wurde erhoben (Zusatzmaterial online: eTabelle 2). Der Suchbegriff „Fuß verstaucht“ war mit 914,6 erwarteten Klicks am beliebtesten. Es schließen sich die Begriffe „Sprunggelenk umgeknickt“ mit 910,9 und „Knöchel verstaucht“ mit 837,9 Klicks an. Danach folgte mit 230,7 Klicks „Sprunggelenk verstaucht“ und „Fuß umgeknickt“ mit 148,1 Klicks. Fachspezifische sowie umgangssprachliche Formulierungen waren weniger gebräuchlich. Die genannten Suchbegriffe wurden für das weitere Verfahren festgelegt. Entsprechend der beschriebenen Vorgehensweise wurden am 16.06.2023 je 125 Internetseiten von Google und Bing erfasst. Nach Anwendung der Ausschlusskriterien konnten 77 Online-Ressourcen in die Studie eingeschlossen werden (Abb. 1).

**Abb. 2:**
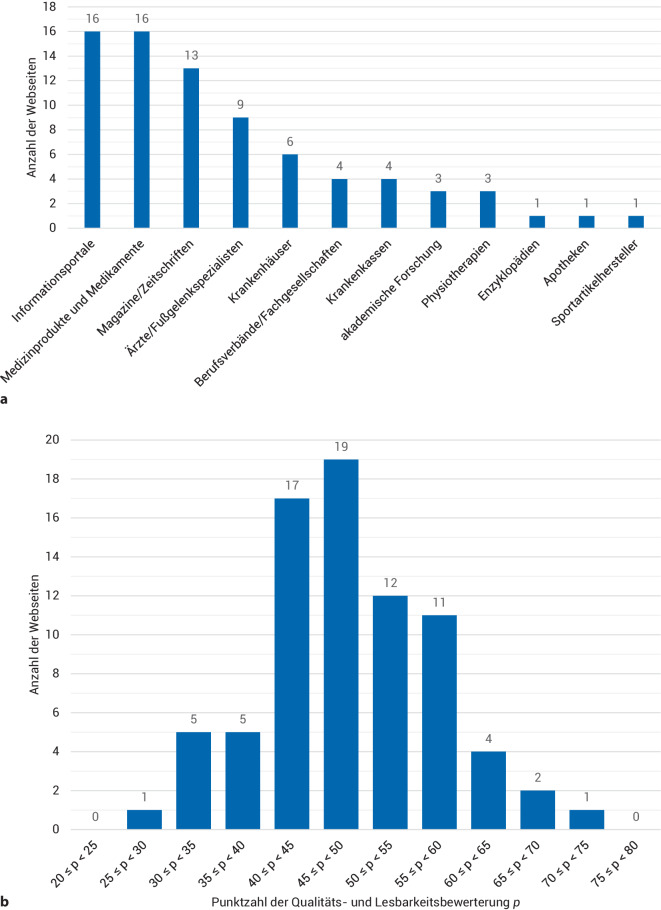
Klassifikation der Webseiten, basierend auf der Urheberschaft (**a**), wobei Informationsportale sowie Webseiten von Medikamenten‑/Medizinproduktanbietern dominierten, sowie die Darstellung der erreichten Gesamtpunktzahl *p* berechnet aus dem 25-Item-Score, dem EQIP36-Score und der Lesbarkeit bei max. 100 erreichbaren Punkten (**b**). Durchschnittlich wurden 48,3 ± 8,6 Punkte erzielt, wobei 77 % im Wertungsbereich zwischen 40 und 60 Punkten lagen. Die Bewertung reichte von 27,7 bis 70,7 Punkten

Am häufigsten waren unabhängige Informationsportale sowie Webseiten von Medikamenten‑/Medizinproduktanbietern vertreten (Abb. 2a). Davon wurden 59 Quellen (77 %) dem Gesundheitswesen zugeordnet und 30 (39 %) als kommerzielle Anbieter erfasst. Mit einer Anzahl von 48 wurden 62 % der Webseiten mit Werbung in Verbindung gebracht, und eine Mehrzahl von 63 (82 %) hatte Verweise zu Social Media.

Im 25-Item-Score wurde eine durchschnittliche Punktzahl von 9,9 ± 4,4 (± Standardabweichung) erzielt, wobei die inhaltliche Vollständigkeit mit Ergebnissen von 1 bis 20 stark variiert [11]. Webseiten, die nicht mit dem Gesundheitssystem assoziiert werden, wiesen einen geringfügig unterdurchschnittlichen Wert von 8,7 ± 4,7 im Vergleich zu Ressourcen aus dem Gesundheitsbereich mit 10,3 ± 4,2 auf. Informationsquellen von kommerziellen Anbietern erzielten mit 8,6 ± 3,6 im Schnitt eine schlechtere Bewertung. Hinsichtlich des Vorhandenseins von Werbung und Links zu Social Media konnte kein Zusammenhang ermittelt werden. Der meistgenannte Fakt bezüglich der ASD war mit 88 % (68/77 Webseiten) die Definition des PECH-Schemas zur Erstversorgung der Verletzung (Zusatzmaterial online: eTabelle 1).

Im EQIP36-Score wurden durchschnittlich 53,0 ± 10,8 bei maximal erreichbaren 100 Punkten erzielt (Zusatzmaterial online: eTabelle 3). Die Kategorie „Inhalt“ schnitt mit 40 % am schlechtesten ab, gefolgt von der Kategorie „Identifizierung“ mit 44 %. Die Webseiten erreichten mit 55 % der möglichen Punkte in der Kategorie „Struktur“ die besten Werte.

Mit einer durchschnittlichen Lesbarkeit von 52,4 ± 9,9 von 100 liegt diese im mittleren Bereich. Es konnten 17 leicht verständliche Webseiten (60 bis 100 Punkte) von 58 mittel gut lesbaren (30 bis 60 Punkte) und 2 schwer lesbaren Quellen (unter 30 Punkte) unterschieden werden. Das Sprachniveau wurde als überwiegend ausgewogen wahrgenommen, bei einer Länge von 13,3 ± 2,3 Wörtern/Satz. Die durchschnittliche Lesedauer sowie die Anzahl der Wörter, der Sätze, der Buchstaben, Zeichen und Absätze schwankten stark (Tab. 1). Für Informationstexte an die allgemeine Bevölkerung wird ein Flesch-Kincaid-Index über 60 empfohlen, was dem Sprachniveau von 13- bis 15-jährigen Schülern der 8. bis 9. Klasse entspricht [26, 29]. Dieses Niveau wurde mit 52,9 ± 6,4 Punkten nur in 14 % (11/77 Webseiten) eingehalten. Bei einem Spearman-Koeffizienten von − 0,246 ergab sich eine negative, monotone Beziehung zwischen der Lesbarkeit und dem inhaltlichen Gehalt. So wiesen Ressourcen, die nicht mit dem Gesundheitssystem assoziiert sind, mit 57,3 ± 10,1 eine leicht überdurchschnittliche Lesbarkeit auf.

**Tab. 1:** Auswertung der Textanalyse der Webseiten

Kriterien der Textanalyse	Mittelwert	Standardabweichung	Median	Interquartilsabstand
Lesbarkeit	52,4	±9,9	53,0	11,0
Wörter	1123,0	±1063,5	894,0	587,0
Sätze	96,7	±108,6	71,0	45,0
Wörter pro Satz	13,3	±2,3	13,2	2,6
Flesch-Index	52,9	±6,4	53,0	8,0
Lesezeit (in min)	05:07	±04:50	04:04	02:41
Buchstaben	7096,8	±6970,8	5496,0	3605,0
Zeichen	8282,8	±8116,4	6467,0	4131,0
Absätze	43,3	±46,8	30,0	19,0

Bei durchschnittlich 48,3 ± 8,6 Punkten im Gesamtranking befinden sich 77 % der Webseiten im Wertungsbereich zwischen 40 und 60 Punkten (Abb. 2b). Die 3 am besten bewerteten waren von der *Apothekenumschau* [33], der Techniker Krankenkasse [34] und IQWiG-Gesundheitsinformationen [35].

**Abb. 3:**
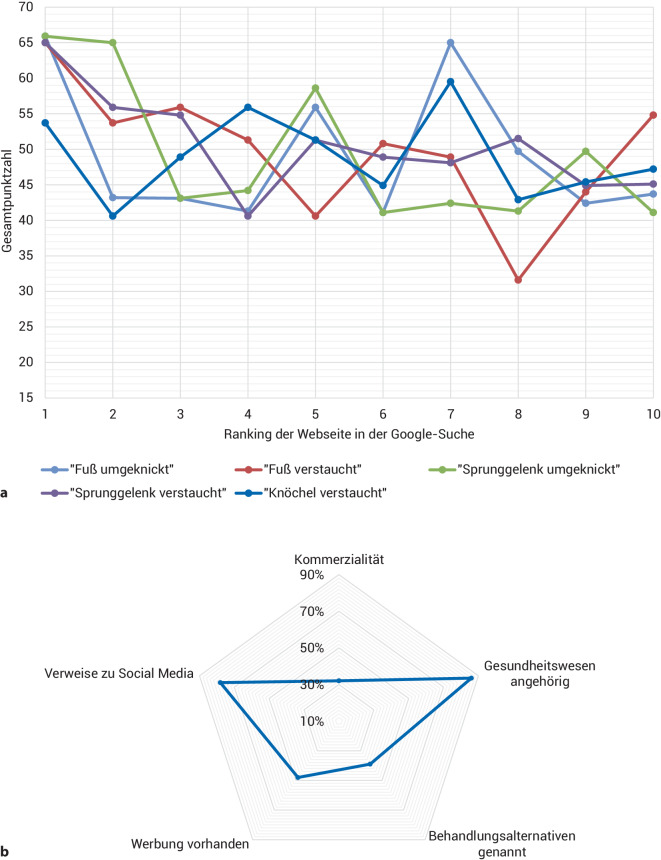
Die Darstellung der Gesamtpunktzahlen der ersten 10 Webseiten innerhalb der Google-Suche, nach Suchbegriffen aufgeschlüsselt. (**a**) Die am besten bewertete Webseite erscheint im Suchalgorithmus bei 4 von 5 Suchbegriffen als Erste. Die weiterführende Klassifikation dieser je ersten 10 Webseiten (**b**) legt eine überwiegende Zuordnung zum Gesundheitswesen sowie das gehäufte Auftreten von Verweisen zu Social Media dar

Neben einer großen Varianz der Werte konnte in 4 von 5 Fällen beobachtet werden, dass die Webseite mit der höchsten Qualität unter den ersten 10 vorgeschlagenen Suchergebnissen als oberste in der Suche aufgelistet wurde und eine überdurchschnittlich hohe Punktzahl im Gesamt-Ranking erhielt (Abb. 3a). Von diesen ersten 10 Webseiten war die Mehrheit dem Gesundheitssystem zugehörig (86 %) und wies Verweise zu Social Media (78 %) auf (Abb. 3b). Der Anteil der Werbung hält sich mit 48 % im Mittelfeld auf, wobei 32 % auf Gewinn ausgerichtet sind.

**Abb. 4:**
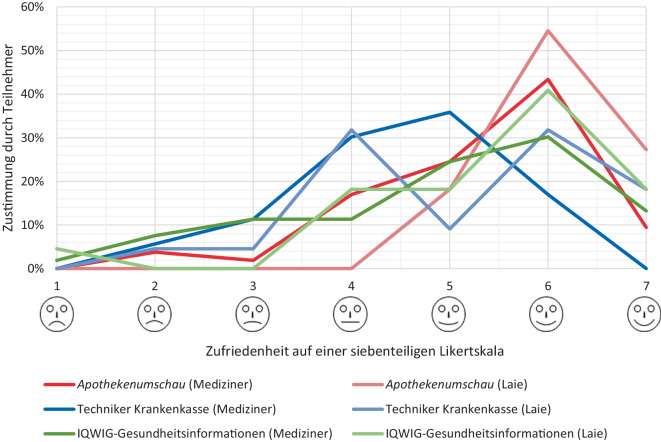
Auswertung der Umfragezufriedenheit mit den Webseiten auf einer 7‑stufigen Likert-Skala, dargestellt für 2 Gruppen an Teilnehmern – jene mit medizinischem Vorwissen (*dunklere Farbtöne*) und medizinische Laien (*hellere Farbtöne*). Die Webseiten wurden von Teilnehmern mit medizinischem Vorwissen mit 4,9 ± 0,4 deutlich kritischer bewertet als durch Laien mit 5,5 ± 0,5 von 7 Punkten

An der Umfrage nahmen 75 Teilnehmer, von denen 53 ein medizinisches Vorwissen angaben und sich 75 % aufgrund einer eigenen ASD bereits mit dem Thema beschäftigt hatten, teil. Bei ausgeglichener Geschlechterverteilung konnten in allen Altersgruppen von 20 bis 74 Jahren Antworten erfasst werden. Die Umfrage ergab eine durchschnittliche Zufriedenheit der Webseiten von 5,1 ± 0,4 auf einer 7‑stufigen Likert-Skala, wobei die in dieser Arbeit am besten bewertete Webseite auch in der Umfrage mit einem Wert von 5,5 (79 %) die höchste Zufriedenheit erlangte [19]. Während die Webseiten von Laien mit 5,5 ± 0,5 bewertet wurden, waren Teilnehmer mit medizinischem Vorwissen mit 4,9 ± 0,4 deutlich kritischer (Abb. 4).

## Diskussion

Die analysierten Internetseiten variieren untereinander sehr und stellen eine repräsentative Stichprobe der aktuell vorhandenen Online-Informationsquellen dar. Der 25-Item-Score zeigt große inhaltliche Unvollständigkeiten. Keine Webseite beinhaltete alle erforderlichen Fakten. Im EQIP36-Score wurde festgestellt, dass eine Großzahl der bereitgestellten Informationen nicht ausreichend strukturiert und die Transparenz zur Herkunft dieser nicht gegeben war. Bei einer durchschnittlich mittleren Lesbarkeit wurde das empfohlene Leseniveau nur in 14 % erreicht. Somit zeigt die durchgeführte Analyse deutlich, dass vorhandene Online-Ressourcen zur ASD Mängel in Bezug auf die Qualität des Inhalts, der Lesbarkeit und der Struktur aufweisen. Dies schränkt die effektive Nutzung durch Patienten ein und kann sich negativ auf die Selbstwirksamkeit auswirken. Obwohl keine der untersuchten Webseiten nahe an die mögliche Gesamtpunktzahl herankam, erwies sich die Zufriedenheit in der Umfrage größer als erwartet. Dies lässt die Frage offen, ob vorhandene Online-Ressourcen als Gesamtbild nicht grundsätzlich schlecht sind oder Patienten nichts Besseres gewohnt sind. Es ist für Patienten möglich, sich umfassend zum Thema zu informieren. Jedoch benötigt es dazu mehr als eine Quelle, was ein Hindernis darstellt.

### Limitationen.

Diese Webanalyse stellt eine Momentaufnahme dar, wobei außerhalb dieser Rahmenbedingungen andere gute Internetseiten existieren können. Eine Änderung der Ergebnisse in naher Zukunft ist möglich. Der Vergleich mit ähnlichen Studien der vergangenen Jahre lässt allerdings einen gleichbleibenden Mangel an qualitativ hochwertigen Gesundheitsinformationen im Internet vermuten [2, 6, 11, 12, 26, 27].

## Ausblick

Mit einem Altersgipfel zwischen 15 und 19 Jahren für die ASD ist die hauptsächlich betroffene Population sehr jung. Dies kann zu einem Wandel in der Art und Weise, wie man sich im Privaten informiert, führen. Auch wenn Videos in dieser Arbeit ausgeschlossen wurden, so konzentrieren sich neue Studien auf diese [28, 30].

Auf Grundlage dieser Ergebnisse wurde die Webseite Kompetenznetzwerk Fuß erstellt [31], die die hier aufgestellten Anforderungen bestmöglich erfüllt. Sie wurde um Fotos und Videos ergänzt, die Patienten helfen sollen, eigenständig im häuslichen Umfeld Stärkungsübungen für das Sprunggelenk durchzuführen. Nach dem hier verwendeten Bewertungssystem erzielt die Webseite mit einem vollständig erfüllten 25-Item-Score und EQIP36-Score sowie einer leichten Lesbarkeit von 69 einen Wert von 90 von 100 Punkten. Dabei entspricht die Lesbarkeit bei einem Flesch-Kincaid-Score von 60 ebenfalls dem empfohlenen Leseniveau und konnte mithilfe des vorherig erwähnten KI-basierten Textanalyse-Tools erzielt werden. Eine höhere Punktzahl war bei Erhalt der inhaltlichen Vollständigkeit schwer möglich. Dennoch wurde die hier erstellte Webseite im Vergleich zur nächstbesten Internetseite mit 71 Punkten deutlich besser bewertet.

## Fazit für die Praxis

- Die Qualität von Webseiten über Sprunggelenkdistorsionen variiert erheblich, wobei keine alle notwendigen Anforderungen erfüllt.
- Eine mangelhafte Qualität und erschwerte Lesbarkeit schränken die effektive Nutzung von Webseiten ein.
- Herausgeber von digitalen Gesundheitsinformationen sollten diese Mängel berücksichtigen, um die Selbstwirksamkeit der Patienten zu steigern und Langzeitergebnisse zu verbessern.

## Supplementary Information

Zusätzliche Tabellen und Fragebogen der Benutzerumfrage

## Data Availability

Die erhobenen Datensätze können auf begründete Anfrage in anonymisierter Form beim korrespondierenden Autor angefordert werden.
