# Supplementary material for: Online resources for ankle sprains: A German language web analysis
Source: Unfallchirurgie (Heidelb). 2025 Jan 16;128(3):201–8. [Article in German] doi: 10.1007/s00113-024-01526-x (PMC11850430; doi:10.1007/s00113-024-01526-x)
Supplement: Supplementary file 1 — Zusätzliche Tabellen und Fragebogen der Benutzerumfrage [file 113_2024_1526_MOESM1_ESM.pdf]

Online-Ressourcen zu Sprunggelenksdistorsionen

Weiterführendes Material

Sophie M. Tengler, apl. Prof. Dr. med. habil. Mark Lenz, Univ.-Prof. Dr. med. Dr. rer. nat. Gunther O. Hofmann, Marianne Rosenthal, PD Dr. med. Klaus E. Roth, Lena Mohr, Prof. Dr. med. Hazibullah Waizy, apl. Prof. Dr. med. habil. Kajetan Klos

eTabelle 1: Auswertung des 25-Item-Scores.

**Kriterien des 25-Item-Scores**	**Anzahl Webseiten**	**Anteil (in %)**
Anatomie des Sprunggelenks beschrieben (Knochen)	15	19,5
Aufbau des Bandapparates beschrieben	19	24,7
Symptome einer Sprungelenksdistorsion	65	84,4
Unfallhergang der Sprunggelenksdistorsion (Supinationstrauma)	40	51,9
Bedeutung als sehr häufige Sportverletzung	45	58,4
Prädispositionen: schwache Bänder, Hohlfuß, Rückfußvarus, Muskelschwäche, Sportler	17	22,1
Erstversorgungsmaßnahmen (PECH-Schema)	68	88,3
Körperliche Untersuchung durch den Arzt (Inversionsstresstest, Schubladentest)	18	23,4
Gradeinteilung der akuten Sprunggelenksdistorsion (drei Stadien)	24	31,2
Indikationen zur Anordnung eines Röntgens durch den Arzt	41	53,2
Indikationen zur Anordnung eines MRT	31	40,3
Begleitverletzungen (Frakturen, Knorpelschäden, Sehnenverletzung, Syndesmosenverletzung)	41	53,2
Konservative Behandlung mit Schiene/Orthese/Bandage	54	70,1
Erklärung der Orthesenbehandlung (Tag und Nacht zu tragen)	4	5,2
Zeitraum der Orthesenbehandlung	24	31,2
Notwendigkeit von Gehstützen	16	20,8
Physiotherapie zur Rehabilitation	38	49,4
Komplikationen der konservativen Therapie (chronisch instabiles Sprunggelenk erwähnt)	38	49,4
Spätfolgen eines chronisch instabilen Sprunggelenks (wiederkehrende Verstauchungen, Knorpelschäden, Arthrose)	23	29,9
Empfehlung des (erneuten) Arztbesuches bei Ausbleiben einer Besserung	22	28,6
Operationsindikationen	22	28,6
Empfehlung zu einer initial konservativen Therapie erwähnt	36	46,8
Wann ist mit der Rückkehr in den Alltag oder zu sportlichen Aktivitäten zu rechnen? (voraussichtlicher Zeitraum)	32	41,6
Prophylaxe gegen ein erneutes Umknicken des Fußes	38	49,4
Notwendigkeit der Thromboseprophylaxe bei Entlastung	5	6,5

eTabelle 2: Prognose ausgewählter Suchbegriffe für Juni 2023 von Google Ads.

Suchbegriff	Geschätzte Klicks	Geschätzte Impressionen
Bänderdehnung Sprunggelenk	46,7	202,8
Bänderriss Sprunggelenk	28,0	134,8
Distorsion Sprunggelenk	2,7	17,1
Fuß umgeknickt	148,1	832,8
Fuß verstaucht	914,6	3645,7
Knöchel umgeknickt	1,2	5,7
Knöchel verstaucht	837,9	3310,9
Knöchel vertrampt	0,0	0,0
Sprunggelenk umgeknickt	910,9	3913,9
Sprunggelenk verdreht	0,0	0,0
Sprunggelenk verknickt	0,0	0,0
Sprunggelenk verstaucht	230,7	1092,3
Sprunggelenkszerrung	0,0	0,0
Umknicktrauma Fuß	0,0	0,0
Umknickverletzung Knöchel	0,0	0,0

eTabelle 3: Auswertung des EQIP36-Scores.

Kriterien des EQIP36-Scores	Anzahl Webseiten	Anteil (in %)
Initiale Definition, welche Themen abgedeckt werden	71	92,2
Abdeckung der oben definierten Themen (wenn bei Q1 = „nein“, dann bei Q2 „nicht enthalten“)	70	90,9
Beschreibung des medizinischen Problems	61	79,2
Definition des Zwecks des medizinischen Eingriffs	58	75,3
Beschreibung der Behandlungsalternativen (einschließlich „keine Behandlung“)	30	39,0
Beschreibung des Ablaufs des medizinischen Verfahrens Wenn „ja“: Vor, während und nach der Intervention	28	36,4
Beschreibung der qualitativen Vorteile	6	7,8
Beschreibung des quantitativen Nutzens (Prozentzahlen)	3	3,9
Beschreibung der qualitativen Risiken und Nebeneffekte	38	49,4
Beschreibung der quantitativen Risiken und Nebenwirkungen (Prozentzahlen)	8	10,4
Behandlung von Fragen der Lebensqualität (trifft eventuell bei kurzen Interventionen nicht zu)	30	39,0
Beschreibung, wie mit möglichen Komplikationen umgegangen wird	24	31,2
Beschreibung der Vorsichtsmaßnahmen, die der Patient ergreifen kann	41	53,2
Erwähnung von Warnzeichen, die der Patient erkennen kann	4	5,2
Auseinandersetzung mit den Kosten medizinischer Interventionen und der Versicherungsproblematik	2	2,6
Spezifische Kontaktinformationen für Krankenhausdienstleistungen	16	20,8

Spezifische Angaben zu anderen Quellen zuverlässiger Informationen/Unterstützung	66	85,7
Das Dokument behandelt alle relevanten Fragen zum Thema (Zusammenfassend für alle inhaltlichen Kriterien).	0	0,0
Datum der Ausgabe oder Erarbeitung	48	62,3
Logo der ausstellenden Stelle	76	98,7
Namen der Personen oder Einrichtungen, die das Dokument erstellt haben	40	51,9
Namen der Personen oder Einrichtungen, die das Dokument finanziert haben	11	14,3
Kurze Bibliographie der im Dokument verwendeten evidenzbasierten Daten	25	32,5
Das Dokument gibt an, ob und wie die Patienten bei seiner Erstellung einbezogen/konsultiert wurden.	1	1,3
Verwendung der Alltagssprache, Erklärung komplexer Wörter oder des Fachjargons	74	96,1
Verwendung von Generikanamen für alle Medikamente und Medizinprodukte	27	35,1
Verwendung kurzer Sätze (im Durchschnitt < 15 Wörter pro Satz)	64	83,1
Das Dokument richtet sich persönlich an den Leser.	47	61,0
Der Ton ist respektvoll.	62	80,5
Die Informationen sind klar (keine Zweideutigkeiten oder Widersprüche).	70	90,9
Informationen werden zwischen Risiken und Nutzen abgewogen.	6	7,8
Die Informationen werden in einer logischen Reihenfolge präsentiert.	68	88,3
Die Gestaltung und das Layout sind zufriedenstellend (mit Ausnahme von Abbildungen oder Graphiken).	72	93,5
Die Zahlen und Diagramme sind klar und aussagekräftig (falls nicht vorhanden = „Nicht zutreffend“)	16	20,8
Das Dokument enthält ein Feld für Notizen des Lesers.	5	6,5
Das Dokument enthält eine Einverständniserklärung.	0	0,0

Benutzerumfrage zu Webseiten über die Sprunggelenksdistorsion:

- Wie alt sind Sie? (Freitext)
- Zu welchem Geschlecht fühlen Sie sich angehörig?
    - Weiblich
    - Männlich
    - Divers
- Welches ist der höchste Bildungsabschluss, den Sie haben?
    - Noch Schüler
    - Schule beendet ohne Abschluss
    - Hauptschulabschluss/Volksschulabschluss
    - Realschulabschluss (Mittlere Reife)
    - Abschluss Polytechnische Oberschule 10. Klasse (vor 1965: 8. Klasse)

    - Fachhochschulreife (Abschluss einer Fachoberschule)
    - Abitur, allgemeine oder fachgebundene Hochschulreife (Gymnasium bzw. EOS)
    - Hochschulabschluss
    - Anderer Schulabschluss (Freitext)
- Gehören Sie einer der folgenden Gruppen an?
    - Arzt/Ärztin
    - Medizinstudent*in
    - Krankenpfleger*in
    - Ergotherapeut*in
    - Physiotherapeut*in
    - Heilpraktiker*in
    - Keine
- Welche Informationsquellen nutzen Sie als Erstes im Falle einer Erkrankung?
    - Internet (z.B. Suchmaschinen wie Google und Bing)
    - Fachliteratur
    - Bekanntenkreis
    - Arztbesuch
    - Social Media
    - Artificial intelligences (wie z.B. ChatGPT)
    - Andere
- Sind Sie schon einmal mit dem Fuß umgeknickt und haben sich das Sprunggelenk verstaucht?
    - Ja
    - Nein

    Falls Sie mit "Nein" geantwortet haben, so gehen Sie bitte direkt zur nächsten Seite weiter und lassen die folgenden Fragen auf dieser Seite unbeantwortet.

- Welche Maßnahmen zur Versorgung und Schmerzlinderung haben Sie selbst ergriffen?
    - Durchführung der PECH-Regel (Pause, Eis, „C“ompression, Hochlagern)
    - Einnahme von Schmerzmedikamenten
    - Anwendung von Bandagen, Orthesen oder Tape
    - Verwendung von Gehhilfen
    - Keine
    - Andere
- Sind Sie zum Arzt gegangen?
    - Ja
    - Nein
- Leiden Sie unter anhaltenden Beschwerden hinsichtlich der Stabilität des Sprunggelenks? (Beispiele sind ein häufiges Umknicken des Knöchels, das Gefühl des Nachgebens und der Instabilität der Bänder oder andauernde Schmerzen im Knöchel)
    - Ja
    - Nein
    - Ich litt nach einer Knöchelverstauchung darunter, aber jetzt nicht mehr.
- Wie oft pro Woche betreiben Sie Sport?
    - Nicht
    - 1 Mal

    - 2 Mal
    - 3 Mal
    - Mehr als 3 Mal

  Wenn Sie die vorherige Frage mit "Nicht" beantwortet haben, bitte ich Sie direkt zur nächsten Seite weiterzugehen und die folgenden Fragen auf dieser Seite unbeantwortet zu lassen.

  Falls Sie eine der anderen Antwortmöglichkeiten gewählt haben, so fahren Sie bitte mit den folgenden Fragen wie gehabt fort.

- Wie lang ist hierbei eine Trainingseinheit?
    - Bis 30 Minuten lang
    - Zwischen 30 Minuten und einer Stunde
    - Länger als eine Stunde
- Welche Sportarten betreiben Sie?
    - Ausdauersport
    - Kraftsport
    - Kampfsport
    - Technisch-akrobatischer Sport (wie z.B. Turnen, Eiskunstlauf und Tanz u.a.)
    - Spielsport (Sportspiele wie z.B. Fußball, Volleyball, Basketball usw.)

- Ich bitte Sie nun die folgende Webseite einmal durchzulesen. Nachdem Sie dies getan haben, können Sie auf die nächste Seite der Umfrage blättern und die Fragen zu dieser beantworten. → Webseite 1
- Welchen Eindruck haben sie nach dem Lesen der Webseite? Folgende Aussagen konnten mit „stimme nicht zu“, „stimme teilweise zu“, „stimme überwiegend zu“ und „stimme zu“ beantwortet werden
    - Die Webseite beschreibt die Symptome der akuten Sprunggelenksdistorsion zutreffend.
    - Die Bilder und der Text tragen zum Verständnis von Anatomie und Funktion des Bandapparates sowie zum Unfallhergang der Sprunggelenksdistorsion bei.
    - Der Aufbau der Webseite ist logisch und nachvollziehbar.
    - Medizinische Fachwörter wurden verständlich erklärt.
    - Ich weiß nach Studium der Internetseite, welche ärztlichen diagnostischen und therapeutischen Maßnahmen durchgeführt werden können.
    - Nach dem Lesen der Webseite kann ich Erstversorgungsmaßnahmen selbstständig, ohne weitere medizinische Anleitung durchführen und Hausmittel anwenden.
    - Die weiterführenden Links und Quellen sind ausreichend.
    - Nach dem Lesen der Internetseite ist mir bewusst, bei welcher Symptomatik ich einen Arzt zur weiteren Abklärung konsultieren sollte (z.B. aufgrund möglicher schwerwiegender Begleitverletzungen).
    - Die Webseite wirkt auf mich vertrauenswürdig und ich nehme die mitgeteilten Informationen ernst.
    - Nach dem Studium der Internetseite fühle ich mich umfassend zum Thema informiert.

- Wie bewerten Sie die Webseite insgesamt?
  - Siebenteilige Likertskala mit Visualisierung:
    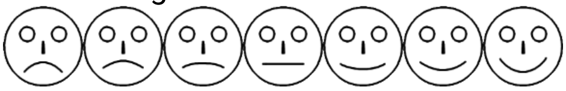
- Wiederholung der webseitenspezifischen Fragen analog für Webseite 2 und 3
- Freitext für Anmerkungen zur Umfrage
